# MULTIDIMENSIONAL SLEEP HEALTH METHODS: FROM THE SIMPLE TO THE SUBLIME

**DOI:** 10.1093/geroni/igae098.0847

**Published:** 2024-12-31

**Authors:** Meredith Wallace

**Affiliations:** University of Pittsburgh, Pittsburgh, Pennsylvania, United States

## Abstract

The multidimensional sleep health framework presents a promising framework for improving sleep – and its health consequences – at a population level. However, there are several methodological challenges to sleep health research. including operationalizing sleep health, incorporating multiple sleep features, domains, and modalities into analytic strategies, organizing differential findings across multiple populations and health outcomes, and clarifying heterogeneity in multidimensional sleep health patterns across individuals. In this presentation, Dr. Wallace will outline several of these important methodological challenges and delineate the advantages and disadvantages of various approaches that can be used to address them. Specifically, she will discuss a range of methods, including methods for sleep data harmonization, weighted and unweighted sleep health composite scores, factor analysis to define sleep dimensionality, clustering to clarify heterogeneity in sleep health patterns, and supervised machine learning to evaluate the extent to which sleep health predicts health outcomes. Finally, Dr. Wallace will demonstrate these methods using an international multi-cohort sample of older adults, including data from the Osteoporotic Fractures in Men Study, the Study of Osteoporotic Fractures, the Multi-Ethnic Study of Atherosclerosis, the Rush Memory and Aging Project, and Minority Aging Research Study, and the Rotterdam Study. With these data and methods, she will discuss the role of sleep health in relation to longitudinal trajectories of depression and cognitive outcomes.

